# Unlocking insights into HIV care: An in-depth analysis of key populations from the Fast-Track Cities Quality of Care survey in eThekwini, KwaZulu-Natal, South Africa

**DOI:** 10.4102/sajhivmed.v27i1.1802

**Published:** 2026-04-21

**Authors:** Somasundram Pillay, Nombulelo Magula, Nceba Gqaleni, Deepak Singh, Zuniga M. Jose

**Affiliations:** 1 Department of Internal Medicine, Faculty of Medicine, University of KwaZulu-Natal, Durban, South Africa; 2 Department of Internal Medicine, Faculty of Medicine, Victoria Mxenge Hospital, Durban, South Africa; 3 Department of Traditional Medicine, Faculty of Medicine, University of KwaZulu-Natal, Durban, South Africa; 4 African Health Research Institute, Faculty of Medicine, University of KwaZulu-Natal, Durban, South Africa; 5 Department of Physics, Durban University of Technology, Durban, South Africa; 6 International Association of Providers in AIDS Care, Washington, United States of America

**Keywords:** HIV, key populations, health service utilisation, non-governmental organisations, urban HIV care, South Africa

## Abstract

**Background:**

Key populations experience disproportionate HIV burdens and structural barriers to care.

**Objective:**

To compare healthcare utilisation, HIV knowledge and barriers between key population groups (KPG) and non-key populations living with HIV in eThekwini, South Africa.

**Methods:**

We analysed the eThekwini Fast-Track Cities Quality of Care survey, a cross-sectional study across 30 high-HIV-burden facilities (April to July 2023). Adults living with HIV completed anonymous questionnaires; groups were compared using *χ*^2^ tests and multivariable logistic regression for partner notification.

**Results:**

Of 517 analysed participants, 128 (24.8%) were KPG. KPG participants were younger, more recently diagnosed (< 1 year: 9.4% vs 1.3%) and more often on antiretroviral therapy (ART) for 1–4 years (52.3% vs 33.4%), with similar daily adherence (~83%). KPG were more likely to access care via non-governmental organisations (NGOs; 23.4% vs 5.7%). Controls more often understood undetectable viral load (36.0% vs 21.9%) and that treatment benefits outweigh side effects (65.3% vs 42.9%). KPG more frequently reported undetectable viral load (55.5% vs 29.8%), more frequent screening for co-morbid conditions, and transport-cost barriers (15.6% vs 8.2%). In KPG, NGO care was associated with partner notification (adjusted odds ratio 18.06; 95% confidence interval 4.77–68.41).

**Conclusion:**

There are marked differences between KPG and adults with HIV in healthcare utilisation, HIV knowledge and structural barriers.

**What this study adds:** Key populations were younger, more recently diagnosed with HIV, and more likely to receive care through NGOs than non-key populations, despite similar ART adherence. Knowledge of undetectable viral load was lower, but screening for comorbidities and reported viral suppression were higher. NGO-based care was independently associated with greater partner notification.

## Introduction

Certain population groups experience a disproportionate burden of HIV. These key populations include men who have sex with men (MSM), people who inject drugs (PWID), sex workers, transgender people, and incarcerated individuals.^1,2^ The magnitude of HIV risk and the determinants driving transmission within these populations vary substantially between high-income countries (HICs) and low- and middle-income countries (LMICs). In South Africa, socioeconomic consequences of HIV and antiretroviral therapy – including impacts on employment and economic participation – have also been demonstrated.^3,4^

MSM remain one of the most heavily affected populations globally. In LMICs, particularly in Africa, HIV prevalence among MSM is amplified by criminalisation, stigma, and social exclusion, which limit access to prevention and treatment services.^5^ In contrast, MSM in many HICs have benefitted from broader access to comprehensive prevention strategies, including pre-exposure prophylaxis (PrEP), contributing to declining HIV incidence in these settings.^6^

PWID constitute another key population with elevated HIV risk. Structural factors such as needle sharing, criminalisation, and inadequate access to opiate substitution therapy continue to drive HIV transmission among PWID in LMICs.^7^ Conversely, in settings where harm-reduction interventions are widely implemented, including needle-exchange programmes and substitution therapy, HIV transmission among PWID has been significantly reduced.^8^

Sex workers experience heightened vulnerability to HIV because of intersecting structural and social determinants, including violence, stigma, criminalisation, and restricted access to healthcare.^9^ Studies from South Africa have reported HIV prevalence estimates ranging from 40% to over 60% in some urban settings, highlighting the disproportionate burden of HIV experienced by this population.^10,11^

Transgender individuals, particularly transgender women, bear an exceptionally high burden of HIV infection. Global estimates indicate that transgender women have nearly 49-fold higher odds of HIV infection compared with the general adult population.^12^ This disparity is further exacerbated in LMICs by limited access to gender-affirming healthcare, pervasive stigma, and discrimination within health systems.^13^

Incarcerated populations also face increased HIV risk resulting from a combination of high-risk behaviours, including injecting drug use and unprotected sexual activity, alongside limited access to HIV prevention and treatment services.^14^ In South Africa, overcrowding and constrained healthcare resources within correctional facilities further compound this risk.^15^

Across these key populations, HIV vulnerability reflects a complex interplay of social, structural, and individual-level factors that vary across socioeconomic and geographic contexts.^2^ Stigmatisation and discrimination remain central barriers to healthcare access, often resulting in delayed diagnosis, suboptimal treatment uptake, and poorer health outcomes.^16^ Discriminatory experiences within healthcare settings can further deter engagement with HIV services.^17,18^ Economic hardship additionally limits access to prevention, testing, and sustained treatment for many individuals in key populations.^19^ Inadequate or inaccurate HIV-related knowledge, frequently driven by misinformation, continues to undermine HIV prevention and care efforts.^20^

Against this background, the present study aimed to compare demographic characteristics, healthcare utilisation patterns, and HIV-related knowledge between key population groups (KPGs) and non-key populations participating in the eThekwini Fast-Track Cities Quality of Care (QoC) survey. We hypothesised that KPGs would demonstrate younger age distributions, greater reliance on non-governmental organisations (NGOs) for healthcare access, and lower understanding of viral-load concepts compared with non-key populations.

Understanding how KPGs living with HIV experience and navigate healthcare services across different socioeconomic and geographic contexts is essential for informing tailored, equitable, and effective HIV interventions.

## Research methods and design

The eThekwini Fast-Track Cities Quality of Care Initiative was carried out as a localised extension of a study spanning 15 Fast-Track Cities, overseen by the International Association of Providers of AIDS Care (IAPAC) within the framework of the Joint UNAIDS-IAPAC Fast-Track Cities Project. This collective effort delivers specialised technical assistance to urban areas heavily impacted by HIV. Among its primary objectives, the project aims to pinpoint and mitigate obstacles in accessing HIV-related care and prevention services by evaluating the perceptions and experiences of PLHIV concerning their care and associated service delivery.

Facilities in the eThekwini Fast-Track City were purposively selected for their high HIV caseloads and their roles in serving key populations, including MSM, sex workers, and PWID. The participating facilities included a combination of public-sector government clinics and hospitals as well as selected NGO facilities providing HIV services, including services specifically targeting key populations. Survey respondents were recruited via convenience sampling of adult PLHIV attending these sites between April and July 2023. Recruitment was conducted by trained research assistants working with clinic staff at participating facilities. Eligible participants were approached in clinic waiting areas and invited to complete the anonymous questionnaire after receiving information about the study.

The present investigation leveraged data from the QoC Survey, which was distributed to consenting PLHIV across 30 healthcare facilities in the eThekwini metropolitan area of KwaZulu-Natal, South Africa, identified as bearing a high HIV burden. To encompass a diverse population, targeted sampling included qualifying categories such as MSM, individuals involved in commercial sex work, and patrons of traditional healthcare practitioners. The focus of the study was to unearth both triumphs and tribulations in the domain of HIV-related care quality, gauging PLHIV’s viewpoints on vital healthcare service metrics and other non-medical determinants.

Adhering to an observational and cross-sectional methodological framework, the study deployed paper-based instruments to conduct anonymous surveys among eligible, consenting PLHIV. The research was executed from April to July 2023. The KPG comprised PLHIV who were 18 years or older, and who self-identified as belonging to one of the following categories:

- Men who have sex with men.
- Commercial sex workers.
- People who inject drugs.
- Migrant.
- Racial ethnic minority (immigrant).
- Lesbian.
- Bisexual.
- Transgender male or transgender female.

For the purposes of this study and based on the small numbers found in the individual sub-groups, the KPG encompassed all of the above sub-groups and was compared to the group of PLHIV who were 18 years and older who did not fall into any one of the abovementioned subgroups (Control).

### Statistical analysis

Numbers and percentages were expressed for categorical variables. Categorical data relationships were determined using the *χ*^2^ test. A *P*-value < 0.05 was used as indicator of significance. *P*-values were not adjusted for multiple comparisons given the exploratory nature of the study. Where logistic regression models were employed, they controlled for age and facility type. Reliability of ordinal scales was assessed using Cronbach’s alpha (approximately 0.84).

### Ethical considerations

Ethical clearance to conduct this study was obtained from the University of KwaZulu-Natal Biomedical Research Ethics Committee (BREC 1750/2020).

## Results

### Study population

A total of 564 individuals completed the QoC survey. Forty-one participants declined to answer questions identifying key population status. Because classification relied on self-reported identity categories, these individuals could not be categorised and were therefore excluded from subgroup analysis. In addition, six were younger than 18 years; these individuals were excluded. The final analytic sample therefore comprised 517 participants, of whom 128 (24.8%) were classified as belonging to a KPG and 389 (75.2%) formed the control group. The composition of the KPG is detailed in Table 1.

**TABLE 1:** Components of the key population group.

Key population group	*n*	%
Men who have sex with men	52	40.6
Lesbian	4	3.1
Bisexual	8	6.3
Commercial sex workers	24	18.8
People who inject drugs	12	9.4
Migrants	12	9.4
Incarcerated	4	3.1
Racial minority	1	0.8
Transgender male	3	2.3
Transgender female	15	11.7

### Demographic and clinical characteristics

KPG participants were significantly younger than the control group, with over-representation in the age group 25–29 years (*P* = 0.03). Recent HIV diagnosis (< 1 year) was more common in the KPG (9.4%) compared with the control group (1.3%; *P* < 0.001). KPG participants were more likely to have been on antiretroviral therapy (ART) for 1–4 years, whereas longer ART duration (> 10 years) was more frequent in the control group (*P* = 0.002). Self-reported ART adherence was high and did not differ significantly between groups. These characteristics are summarised in Table 2.

**TABLE 2:** Demographics of control group versus key population group.

Variables	Control group		KPG		*P*
	*n*	%	*n*	%	
**Age (years)**					
18–24	20	5.1	11	8.6	0.150
25–29	53	13.6	28	21.9	0.030*
30–39	113	29.1	40	31.3	0.640
40–49	143	36.8	35	27.3	0.050*
50–59	44	11.3	12	9.4	0.540
60+	16	4.1	2	1.6	0.170
**Duration of living with HIV (years)**					
< 1	5	1.3	12	9.4	< 0.001*
1–4	122	31.4	52	40.6	0.050
5–9	126	32.4	30	23.4	0.060
10+	136	35.0	34	26.6	0.080
**Duration on ART (years)**					
1–4	130	33.4	67	52.3	0.001*
5–9	129	33.2	36	28.1	0.290
10+	130	33.4	24	18.8	0.002*
Prefer not to answer	0	0.0	1	0.8	0.080
**Current ART compliance**					
Yes, everyday	324	83.5	107	83.6	0.990
Yes, but sometimes miss doses	64	16.5	19	14.8	0.660
Never taken ART	0	0.0	1	0.8	0.080
Prefer not to answer	0	0.0	1	0.8	0.080
**Location where patients receive healthcare from?**					
Government clinic or hospital	385	99.0	94	73.4	< 0.001*
Private clinic	6	1.5	4	3.1	0.260
NGO healthcare provider	22	5.7	30	23.4	< 0.001*
THP	61	15.7	5	3.9	< 0.001*
Other†	3	0.8	0	0.0	0.320

KPG, key population group; ART, antiretroviral therapy; NGO, non-governmental organisation; THP, traditional health practitioner.

* , Denotes significance.

† , Other refers to private pharmacy or a not sure response.

### HIV knowledge, counselling, and perceived treatment challenges

The control group demonstrated greater understanding of the meaning of an undetectable viral load compared with KPG participants (36.0% vs 21.9%; *P* = 0.003). Similarly, a higher proportion of control participants reported understanding that the benefits of HIV treatment outweigh potential side effects (65.3% vs 42.9%; *P* < 0.001). Counselling on HIV transmission by healthcare providers was reported at similarly high levels in both groups. There were no statistically significant differences between groups in reported challenges related to pill burden, dosing frequency, medication side effects, or the social visibility of ART use. These outcomes are shown in Table 3.

**TABLE 3:** Comprehensive analysis of patient-centric health education and challenges encountered in HIV management and intervention.

Variables	Control group *N* = 389		Key population group *N* = 128		*P*
	*n*	%	*n*	%	
**Basic understanding of:**					
VL achieved	95	24.4	21	16.4	0.060
Undetectable VL achieved	140	36.0	28	21.9	0.003*
The need for daily ART compliance	331	85.1	111	88.8	0.650
**Counselling on the spread of HIV infection from:**					
Healthcare provider	368	94.6	121	94.5	0.980
THP	116	29.8	29	22.7	0.120
**Number of patients requesting information on the following:**					
HIV and its treatment	303	77.9	92	71.9	0.160
**The number of patients completely understanding**					
That the benefits of HIV treatment outweigh the side effects	254	65.3	54	42.9	< 0.001*
**Problems faced by patients (agree and somewhat agree responses)**					
The number of pills that I take makes my life difficult	50	12.9	12	9.5	0.710
The number of times that I have to take pills each day make my life difficult.	51	13.1	7	5.5	0.080
I do not like the way the HIV medication makes me look	57	14.6	16	12.6	0.670
The side effects caused by the HIV medications are noticed by other people	75	19.3	21	16.4	0.700
I do not like the way the HIV medication makes me feel	66	17.0	33	25.8	0.180

VL, viral load; ART, antiretroviral therapy; THP, traditional health practitioner; HIV, human immunodeficiency virus.

* , Denotes significance.

### Healthcare utilisation and ART-related care

Patterns of healthcare utilisation differed significantly between the two groups. The control group predominantly accessed HIV care through government clinics or hospitals, whereas KPG participants were significantly more likely to receive care through NGOs and less likely to attend government facilities (*P* < 0.001). Use of private clinics was uncommon in both groups (Table 2).

KPG participants attended health facilities more frequently during the preceding year, with a higher proportion reporting three clinic visits annually (35.2% vs 16.2%; *P* < 0.001). They were also more likely to have undergone two viral load tests in the previous year (44.5% vs 26.5%; *P* = 0.001). ART collection was reported more frequently by the control group, while KPG participants more commonly attended clinics for blood sampling related to viral load testing (*P* < 0.001). A higher proportion of KPG participants reported having an undetectable viral load and fewer reported being unaware of their viral load status (*P* < 0.001). These findings are summarised in Table 4.

**TABLE 4:** Healthcare utilisation, ART care, viral load monitoring, and disclosure.

Variables	Control group		Key population group		*P*
	*n*	%	*n*	%	
**People aware of patient’s HIV status**					
Parents	225	57.8	68	53.1	0.350
Siblings	254	65.3	65	50.8	0.003
Children	181	46.5	37	28.9	< 0.001*
Friends	189	48.6	48	37.5	0.030*
Spouse	156	40.1	17	13.3	< 0.001*
Sexual partners	55	14.1	18	14.1	0.990
Community members	62	15.9	10	7.8	0.020*
Religious leader	51	13.1	2	1.6	< 0.001*
Co-workers	46	11.8	7	5.5	0.040*
Employer	21	5.4	4	3.1	0.300
No one	11	2.8	4	3.1	0.860
**Time from HIV diagnosis to first clinic visit**					
Same day	221	56.8	83	64.8	0.110
< 7 days	56	14.4	15	11.7	0.450
7 days – 1 month	32	8.2	11	8.6	0.900
1–3 months	24	6.2	5	3.9	0.330
> 3 months	56	14.4	14	10.9	0.320
**Time from diagnosis to ART initiation**					
Same day	203	52.2	73	57.0	0.340
< 7 days	61	15.7	24	18.8	0.420
7 days – 1 month	30	7.7	6	4.7	0.240
1–3 months	28	7.2	9	7.0	0.950
> 3 months	67	17.2	14	10.9	0.090
**Clinic visits in previous year (number of times)**					
Never	7	1.8	2	1.6	0.860
1	34	8.7	11	8.6	0.960
2	30	7.7	18	14.1	0.030*
3	63	16.2	45	35.2	< 0.001*
6	160	41.1	32	25.0	0.001*
12	71	18.3	6	4.7	< 0.001*
> 12	24	6.2	14	10.9	0.070
**Viral load monitoring**					
Undetectable	116	29.8	71	55.5	< 0.001*
Detectable	90	23.1	24	18.8	0.300
Unknown	183	47.0	33	25.8	< 0.001*

ART, antiretroviral therapy; HIV, human immunodeficiency virus.

* , Denotes significance.

### Disclosure patterns and patient experience

Disclosure of HIV status to family members, partners, and social contacts was consistently higher in the control group. Statistically significant differences were observed for disclosure to siblings, children, spouses, friends, community members, religious leaders, and co-workers (all *P* ≤ 0.04). Rates of non-disclosure did not differ significantly between groups (Table 4).

Approximately one-third of participants in both groups reported that clinic staff were unfriendly, rude, or unwelcoming. There were no significant differences between the groups regarding negative experiences with healthcare providers or administrative staff (Table 5).

**TABLE 5:** Health education, screening, patient experience, and financial barriers.

Variables	Control group		Key population group		*P*
	*n*	%	*n*	%	
**Education provided by clinic**					
Safe sexual practices	289	74.3	97	75.8	0.740
Harm reduction	205	52.7	53	41.4	0.030*
Partner notification	192	49.4	60	46.9	0.630
PMTCT	142	36.5	26	20.3	< 0.001*
None	81	20.8	21	16.4	0.280
**Screening performed**					
Depression	25	6.4	16	12.5	0.030*
Hepatitis C	8	2.1	20	15.6	< 0.001*
Hepatitis B	6	1.5	29	22.7	< 0.001*
TB screening	159	40.9	91	71.1	< 0.001*
Diabetes mellitus	117	20.1	64	50.0	< 0.001*
Hypertension	139	35.7	68	53.1	< 0.001*
Sexually transmitted infections	19	4.9	31	24.2	< 0.001*
None	164	42.2	10	7.8	< 0.001*
**Patient experience**					
Clinic staff unfriendly	121	31.1	36	28.1	0.520
**Financial barriers**					
Transport cost a problem	32	8.2	20	15.6	0.020*
Never a problem	347	89.2	101	78.9	0.003*

PMTCT, prevention of mother-to-child transmission; TB, tuberculosis.

* , Denotes significance.

### Screening for co-morbidities and financial barriers

KPG participants were more frequently screened for a range of co-morbid conditions, including hepatitis B, hepatitis C, tuberculosis, diabetes mellitus, hypertension, sexually transmitted infections, depression, and cervical cancer (all *P* ≤ 0.004). Tuberculosis testing rates were high and comparable between the two groups. These screening patterns are presented in Table 5.

Financial barriers to accessing care were reported more frequently by KPG participants, particularly with respect to transport costs. KPG participants were more likely to report that transport-related expenses sometimes or often limited their ability to attend healthcare services (*P* = 0.003), although most participants in both groups reported that financial constraints rarely or never prevented access to care (Table 5).

### Traditional health practitioner utilisation

Overall, KPG participants were less likely to have visited a traditional health practitioner (THP) in the preceding year compared with the control group (71.1% vs 59.4%; *P* = 0.02). Among those who did attend THPs, KPG participants were more likely to do so for ART collection (*P* = 0.004), whereas the control group more frequently reported visits for other health concerns, viral load discussions, and ART adherence education (*P* ≤ 0.04). Perceived benefit from THP services differed between groups, with control participants more often reporting feeling better ‘some of the time’ or ‘most of the time’ following THP visits. These findings are shown in Table 6.

**TABLE 6:** Analysis of traditional health practitioner visits.

Variables	Control group		Key population group		*P*
	*n*	%	*n*	%	
**Number of times patient visited THP in the last year:**					
Never	230	59.4	91	71.1	0.020
1	22	5.7	11	8.6	0.250
2	41	10.6	5	3.9	0.020*
3	31	8.0	8	6.3	0.510
6	29	7.5	6	4.7	0.270
12	10	2.6	3	2.3	0.880
> 12	24	6.2	4	3.1	0.180
**Reasons for THP visit was to talk about:**					
HIV-related symptoms	50	12.9	9	7.0	0.070
CD4 count	29	7.5	6	4.7	0.280
Viral load	33	8.5	4	3.1	0.040
HIV treatment	44	11.3	9	7.0	0.170
Any other health-related concerns	114	29.3	22	17.2	0.010*
Collect ART	1	0.3	4	3.1	0.004
Collect other medications	36	9.3	10	7.8	0.620
**Education provided by THP:**					
ART compliance	122	31.4	25	19.5	0.010*
Viral load target	74	19.3	17	13.4	0.130
**Process that followed education on partner notification:**					
Partner brought in for testing and counselling	76	19.7	22	17.3	0.550
Partner contact information given to clinic so that clinic could liase with partner directly	22	5.7	4	3.1	0.250
Did not refer or provide any partner information to clinic	120	31.2	28	22.1	0.050
**Does the HIV and health service provided by THP make patient feel better about themselves?**					
Not at all	165	42.4	51	39.8	0.610
A little bit	29	7.5	8	6.3	0.650
Some of the time	31	8.0	2	1.6	0.010*
Most of the time	34	8.7	4	3.1	0.030*
Yes	56	14.4	15	11.7	0.450

THP, traditional health practitioner; ART, antiretroviral therapy; HIV, human immunodeficiency virus.

* , Denotes significance.

### Partner notification and facility type

Among KPG participants, attendance at NGO healthcare facilities was strongly associated with partner notification. In multivariable analysis adjusting for age and facility type, receiving HIV care through an NGO was associated with significantly increased odds of partner notification (adjusted odds ratio 18.06; 95% confidence interval 4.77–68.41; *P* < 0.001). No significant associations were observed for government clinics, private clinics, or THPs. These results are presented in Table 7.

**TABLE 7:** Key population group and partner notification.

Variables	Bivariate analysis				Multivariate analysis		
	Unadjusted OR	95% CI	Pearson Chi-square value	*P*	Adjusted OR	95% CI	*P*
Government clinic or hospital	3.714	1.275–10.815	0.025	0.874	1.242	0.342–4.503	0.742
Private clinic	2.222	0.495–9.969	0.006	0.937	1.136	0.231–5.580	0.876
NGO healthcare provider	6.009	2.312–15.621	10.464	0.001	18.055	4.765–68.412	< 0.001
Traditional health practitioner	0.740	0.403–1.360	0.760	0.383	0.584	0.301–1.136	0.113

OR, odds ratio; CI, confidence interval; NGO, non-governmental organisation.

Technical note: Denominators vary across analyses as a result of item-level non-response.

## Discussion

This study provides a nuanced assessment of healthcare utilisation patterns, HIV-related knowledge, and service experiences among KPG compared with controls within the eThekwini Fast-Track Cities QoC survey. Although self-reported ART adherence was comparable across groups, substantial differences were observed in service access pathways, understanding of viral load concepts, and exposure to structural barriers, underscoring persistent inequities affecting marginalised populations.

KPG participants were significantly more likely than non-key populations to access HIV care through NGOs. However, it is important to note that the majority of participants in both groups still accessed care through government healthcare facilities. This pattern is consistent with evidence demonstrating the critical role of NGOs in delivering HIV services to marginalised populations, particularly in contexts where public-sector healthcare is perceived as inaccessible or stigmatising.^21^ Historical and structural challenges within South Africa’s health system, including inequitable resource distribution and variable service quality, have long shaped healthcare-seeking behaviour and may contribute to this preference.^3^

The strong association between NGO utilisation and higher partner-notification rates observed in this study further highlights the effectiveness of civil society-led and community-based service models. NGOs often employ peer-led approaches, flexible service delivery, and rights-based frameworks that foster trust and engagement among populations facing discrimination in formal healthcare settings.^21,22^ These findings support calls for stronger integration of NGO-led models into national HIV programmes, rather than positioning them as parallel or peripheral service providers.

Additional findings provide insight into engagement with HIV care among key populations. Participants within the KPG were more recently diagnosed with HIV and had shorter durations of ART exposure compared with controls. Despite similar self-reported adherence, key population participants demonstrated significantly lower understanding of viral load concepts, including the meaning of an undetectable viral load. This finding highlights the need for strengthened counselling and health-literacy interventions within HIV care programmes, targeting key populations.

Interactions with THPs revealed additional complexity in healthcare-seeking behaviour. While overall THP utilisation was lower among KPG participants, this group was more likely to engage THPs for ART collection. Collaboration between THPs and biomedical HIV services has been advocated as a strategy to improve access and continuity of care in sub-Saharan Africa.^23^ However, the effectiveness of such collaboration depends on adequate training, clear referral pathways, and mutual trust between traditional and biomedical sectors.

The lower proportion of KPG participants receiving ART adherence education from THPs compared with the control group suggests potential communication barriers or reluctance to disclose stigmatised identities. Qualitative work from South Africa has highlighted that THPs may experience uncertainty, limited training, or discomfort when engaging with HIV care in rapidly evolving treatment landscapes.^24^ Nevertheless, when appropriately trained and supported, THPs can positively influence ART adherence and patient understanding of HIV treatment goals, including viral load suppression.^25,26^ These findings point to an opportunity for structured engagement and capacity-building of THPs within the broader HIV care continuum.

The younger age profile observed among KPG participants has important implications for HIV prevention and care. Younger individuals living with HIV often face heightened psychosocial vulnerability, including unstable employment, mobility, and social marginalisation, which can disrupt sustained engagement in care.^11^ Mental health comorbidities such as depression and anxiety are common in this population and are known to adversely affect ART adherence and retention in care.^27^ Integrating routine mental health screening and support into HIV services may therefore be particularly beneficial for younger KPG individuals.

Across both groups, a substantial proportion of participants reported negative experiences with healthcare staff, reflecting the ongoing impact of stigma and discrimination within healthcare environments. HIV-related stigma remains a well-established barrier to equitable access to care and is consistently associated with delayed diagnosis, reduced treatment uptake, and poorer health outcomes.^16^ Structural barriers, including transport costs and broader socioeconomic constraints, disproportionately affected KPG participants, further compounding challenges to consistent healthcare engagement.^19^

Although this study did not directly evaluate digital engagement, emerging evidence suggests that digital and social network-based interventions may play an important role in improving HIV knowledge and engagement in care among younger and marginalised populations. Online and social network-based interventions have demonstrated potential in improving HIV knowledge and countering misinformation, particularly among younger and marginalised populations.^28^ Leveraging such approaches alongside facility-based services may strengthen engagement across the HIV care cascade.

In summary, these findings reinforce the need for differentiated, inclusive, and community-informed HIV service delivery models. Strengthened collaboration between government health services, NGOs, THPs, and mental health providers is essential to addressing the intersecting social and structural drivers of inequity and to advancing progress towards national and global HIV targets.

### Limitations

The survey utilised non-random convenience sampling at selected facilities, which may introduce selection bias and limit generalisability. Reliance on self-reported data raises the possibility of recall and social desirability biases. The cross-sectional design precludes causal inference. Moreover, aggregating diverse key populations into a single group may mask subgroup-specific trends. Measures related to feelings (like ‘unwelcoming vibes’ from healthcare staff) are inherently subjective and might vary based on individual thresholds or interpretations. While interactions with THPs were documented, the study might not have captured the depth, nature, or nuances of these interactions, limiting comprehensive understanding. Future research, addressing these limitations can offer more comprehensive insights and robust conclusions. In addition, outcomes such as clinic attendance patterns and reported patient experiences were not stratified by facility type (government vs NGO). As a result, the present analysis cannot determine whether reported negative healthcare experiences occurred within specific facility settings.

## Conclusion

KPGs living with HIV in eThekwini demonstrate distinct patterns of healthcare utilisation, HIV-related knowledge, and structural barriers compared with non-key populations. Although ART adherence was similar between groups, key populations were younger, more recently diagnosed, and more likely to access HIV services through NGOs. These findings highlight the critical role of community-based service models in reaching marginalised populations. Strengthening collaboration between public health services, NGOs, and community providers, while improving HIV literacy and addressing structural barriers such as transport costs, will be essential for achieving equitable HIV care and sustaining progress towards epidemic control.
