# Corrigendum: Unlocking insights into HIV care: An in-depth analysis of key populations from the Fast-Track Cities Quality of Care survey in eThekwini, KwaZulu-Natal, South Africa

**DOI:** 10.4102/sajhivmed.v27i1.1836

**Published:** 2026-05-21

**Authors:** Somasundram Pillay, Nombulelo Magula, Nceba Gqaleni, Deepak Singh, José M. Zúñiga

**Affiliations:** 1 Department of Internal Medicine, Faculty of Medicine, University of KwaZulu-Natal, Durban, South Africa; 2 Department of Internal Medicine, Faculty of Medicine, Victoria Mxenge Hospital, Durban, South Africa; 3 Department of Traditional Medicine, Faculty of Medicine, University of KwaZulu-Natal, Durban, South Africa; 4 African Health Research Institute, Faculty of Medicine, University of KwaZulu-Natal, Durban, South Africa; 5 Department of Physics, Durban University of Technology, Durban, South Africa; 6 International Association of Providers in AIDS Care, Washington, United States of America

In the original article published, Pillay S, Magula N, Gqaleni N, et al. Unlocking insights into HIV care: An in-depth analysis of key populations from the Fast-Track Cities Quality of Care survey in eThekwini, KwaZulu-Natal, South Africa. S Afr J HIV Med. 2026;27(1), a1802. https://doi.org/10.4102/sajhivmed.v27i1.1802, an author’s name was incorrectly spelt.

Instead of:

Zuniga M. Jose

It should be:

José M. Zúñiga

The authors apologises for this error. The correction does not change the study’s findings of significance or overall interpretation of the study’s results or the scientific conclusions of the article in any way.

